# The importance of the clinical classification of adult T-cell leukemia/lymphoma (ATLL) in the prognosis

**DOI:** 10.1371/journal.pntd.0010807

**Published:** 2022-10-19

**Authors:** Pedro Dantas Oliveira, Guilherme Sousa Ribeiro, Rosangela Oliveira Anjos, Maria Almeida Dias, Lourdes Farre, Iguaracyra Araújo, Achiléa Lisboa Bittencourt

**Affiliations:** 1 Department of Internal Medicine, Professor Edgard Santos Teaching Hospital, Federal University of Bahia, Salvador, Bahia, Brazil; 2 Department of Medicine, Federal University of Sergipe, Aracaju, Bahia, Brazil; 3 Laboratory of Pathology and Molecular Biology, Gonçalo Moniz Institute, Oswaldo Cruz Foundation, Salvador, Bahia, Brazil; 4 Department of Preventive and Social Medicine, Federal University of Bahia, Salvador, Bahia, Brazil; 5 Laboratory of Experimental Pathology, Gonçalo Moniz Institute, Oswaldo Cruz Foundation, Salvador, Bahia, Brazil; 6 Program Against Cancer Therapeutic Resistance (ProCURE), Catalan Institute of Oncology (ICO), ONCOBELL, Bellvitge Institute for Biomedical Research (IDIBELL), L’Hospitalet del Llobregat, Catalonia, Spain; 7 Department of Pathology, Professor Edgard Santos Teaching Hospital, Federal University of Bahia, Salvador, Bahia, Brazil; Kumamoto University: Kumamoto Daigaku, JAPAN

## Abstract

**Background:**

Adult T-cell leukemia/lymphoma (ATLL), a peripheral T-cell leukemia/lymphoma associated with the human T-cell lymphotropic virus type-1 (HTLV-1), has been classified following the clinical forms defined by Shimoyama in 1991. A suggestion to modify Shimoyama’s classification was proposed in 2007 to differentiate within the smoldering patients those who presented nodules or tumors in the skin without lung involvement, which was named the primary cutaneous tumoral (PCT) form of ATLL. In the present study, according to their clinicopathological characteristics, we estimated the mortality rates of 143 ATLL patients from Bahia, Brazil. We also evaluated the importance of classifying PCT/ATLL separately from the smoldering type on disease prognosis.

**Methodology/Principal findings:**

Diagnosis of ATLL was established based on a positive serology for HTLV-1, histopathological and/or cytological diagnosis of peripheral T-cell leukemia/lymphoma. Patients were clinically grouped according to Shimoyama’s classification, considering PCT variants separately from the smoldering cases. Bivariate and multivariable survival analyses were applied to identify factors associated with disease prognosis. Significant differences in the median survival time were observed between the clinical types, with the smoldering type presenting the longest median survival (109 months) compared to the other forms (<50 months); the median survival for PCT/ATLL was 20 months. Multivariable analysis confirmed that ATLL clinical types were associated with survival, with a better prognosis for patients with the smoldering and chronic types. Furthermore, skin involvement was related to a worse outcome in the multivariable analysis, regardless of the clinical form and presence of lymphadenopathy.

**Conclusions/Significance:**

Our results reinforce the importance of considering the PCT/ATLL separately from the smoldering type when classifying ATLL to better define prognosis and treatment, given the significant difference in the survival of patients between the smoldering form and PCT/ATLL. Skin involvement should also be considered an independent prognostic factor in patients with ATLL.

## Author summary

The human T-cell lymphotropic virus type-1 (HTLV-1) may cause serious diseases, such as adult T-cell leukemia/lymphoma (ATLL), which can manifest itself in different clinical forms. In this paper, we investigated the survival of 143 Brazilian ATLL patients, according to the clinical form and other clinicopathological characteristics using bivariate and multivariable analyses. The original clinical classification of ATLL was employed; however, we considered the primary cutaneous tumoral (PCT) ATLL and the smoldering type separately. We found statistically significant differences in the mortality rates by clinical types and highlighted the better prognosis of patients with the smoldering type (mortality rate: 0.6 per 100 person-years) compared to those with PCT (mortality rate: 5.3 per 100 person-years). This finding emphasizes the importance of considering the PCT/ATLL separately from the smoldering type when using the ATLL classification to determine prognosis and treatment. PCT/ATLL should be treated with more aggressive ATLL protocols than the smoldering type without skin nodules or tumors.

## Introduction

Adult T-cell leukemia/lymphoma (ATLL) is a peripheral T-cell leukemia/lymphoma associated with the human T-cell lymphotropic virus type-1 (HTLV-1). ATLL is considered an aggressive disease with a poor response to chemotherapy [1,2]. It is more frequently registered in the Southwestern part of Japan, a highly endemic area for HTLV-1 [2,3]. Brazil represents one of the largest HTLV-1 endemic areas in the American continent with a high prevalence of HTLV-1 carriers, as in Japan [2–4]. However, while in Japan there are many reported cases of ATLL, this disease is infrequently detected in Brazil [2,4,5]. It is believed that ATLL is underestimated in Brazil, probably being misdiagnosed with other T-cell lymphomas [4,5].

Shimoyama et al classified ATLL into four clinical types: acute, chronic, lymphoma, and smoldering [6]. The acute and lymphoma forms are considered aggressive diseases, while smoldering and chronic forms are considered indolent [1]. In 2007, Bittencourt et al. [7] suggested including another form of ATLL in Shimoyama’s classification: the primary cutaneous tumoral (PCT). PCT was originally included in the smoldering form [8], but it is a distinct manifestation of ATLL, with cutaneous nodulotumoral lesions [7]. It also has a worse prognosis than the smoldering form and requires a different therapeutic approach [9]. Notwithstanding, the PCT form has not been considered in recent studies on ATLL [10–12].

Previously, we evaluated in the state of Bahia, Brazil, the survival of a cohort of 70 patients with different clinical types of ATLL [7]. Here, with a larger cohort, with twice the number of Brazilian patients, and a longer follow-up, we correlated the different types of ATLL and their clinicopathological characteristics with patient survival using bivariate and multivariable analyses. Patients were classified according to Shimoyama’s classification, and we additionally evaluated the results considering the PCT/ATLL separately from the smoldering type [7].

## Material and methods

### Ethical approval

The Institutional Review Board of the University Hospital of the Federal University of Bahia approved the study protocol (CAAE- 00733818.9.0000.0049). Written informed consent was obtained from all patients before study participation.

### Study population

One hundred and forty-three patients with ATLL were prospectively followed up. They were diagnosed in the Dermatology, Hematology, and Pathology Departments of the University Hospital of the Federal University of Bahia between 1991 and 2017. All patients had positive serology for HTLV-1 and a histopathological and/or cytological diagnosis of peripheral T-cell leukemia/lymphoma. Serology for HTLV-1 was performed by ELISA and confirmed by Western blot or polymerase chain reaction (PCR). All of them were HIV-negative. Seventeen of these patients, all acute or chronic ATLL, were diagnosed hematologically, by blood immunophenotyping and/or by peripheral blood smears. For cases with more prolonged survival or early onset of disease, analyses of clonality using Southern blot [13] or long-inverse PCR [14] were performed. Patients underwent complete physical and dermatological examinations, laboratory tests, including blood count with differential, complete biochemistry, including serum calcium and LDH, peripheral blood smears for evaluation of atypical cells and/or peripheral blood immunophenotyping. Lymphocytosis was defined as the lymphocyte count was > 4 x 10^9^/l. Patients also underwent chest radiography, abdominal and pelvic ultrasonography, and cervical, thoracic and abdominal computed tomography scans. On medical recommendation, a head computed tomography scan was also performed, as well as a cerebrospinal fluid examination. Clinical types were classified at the time of diagnosis, according to Shimoyama’s classification into acute, chronic, lymphoma, and smoldering types [6]. However, within the smoldering type, the cases with nodulotumoral lesions and without lung involvement were here considered as PCT variants and were analyzed separately from the other smoldering cases [7,15]. Nodules and tumors were defined as any solid lesion ≥1cm in diameter with evidence of deep infiltration in the skin (vertical growth) [16]. Some cases in this series participated in previous studies and are now included with a longer follow-up [7,15,17,18].

Structured questionnaires were used to collect epidemiological and clinical data from patients at recruitment and during follow-up. Demographic and clinicopathological data included sex, age (<50 years or ≥50 years of age), clinical type of ATLL, presence of HTLV-1-associated myelopathy/tropical spastic paraparesis (HAM/TSP), lymphadenopathy, hepatomegaly, splenomegaly, involvement of two or more organs, skin involvement, lymphocytosis, high LDH levels, and hypercalcemia. Biopsy was performed in 126 cases (90 of skin, 31 of lymph nodes, and five of other organs) for histopathological and immunohistochemical studies. Bone marrow biopsy was indicated for 99 patients during follow-up (35 acute; 25 lymphomas; 18 smoldering, 15 chronic, and six PCT). The histopathological diagnosis was performed by two pathologists who are experts in hematopathology and dermatopathology (IA and ALB). The ATLL cases were classified histopathologically according to the World Health Organization classification of T-cell leukemias/lymphomas [19]. Immunohistochemical studies were performed with the following markers: CD3, CD4, CD5, CD7, CD8, CD20, CD25, CD30, CD79a, and ALK-1. The proliferative index was assessed by the Ki-67 marker in 118 cases. The following microscopical data were considered in the survival analyses: histopathologic patterns, cell size, and proliferative index.

The treatment consisted of chemotherapy or zidovudine/interferon-α (AZT/IFN-α) or the association of both therapies. Radiotherapy was employed in a few patients with tumoral lesions. Most patients with smoldering ATLL have been managed with active monitoring until disease progression, plus topical cutaneous therapy. Only one patient with acute ATLL was submitted to allogenic stem cell transplantation (allo-HSCT) and is well after three and half years of follow-up. Survival intervals were calculated from the date of diagnosis to the date of death or last follow-up (censoring in October 2018).

### Statistical analysis

We used absolute and relative frequencies to characterize study participants regarding demographics and clinical aspects overall and by clinical type. We calculated median survival times (MST) (and respective interquartile ranges) and mortality rates per 100 person-years of follow-up overall and according to demographic and clinical characteristics. The association between patients’ characteristics and survival was assessed by hazard ratios (HR) and 95% confidence intervals using bivariate and multivariable Cox proportional hazards regression models. We selected variables that had a P value <0.20 in the bivariate analyses for inclusion in the multivariable model, except for bone marrow involvement, lymphocytosis, and hepatomegaly, because they were highly correlated with splenomegaly, number of involved organs, or clinical types. A backward selection method was then employed to build the final multivariable model, which retained variables with a P value <0.05. All data are available in S1 Text and S1 Data.

## Results

### Demographic and clinical characteristics of the ATLL patients

**Fig 1:**
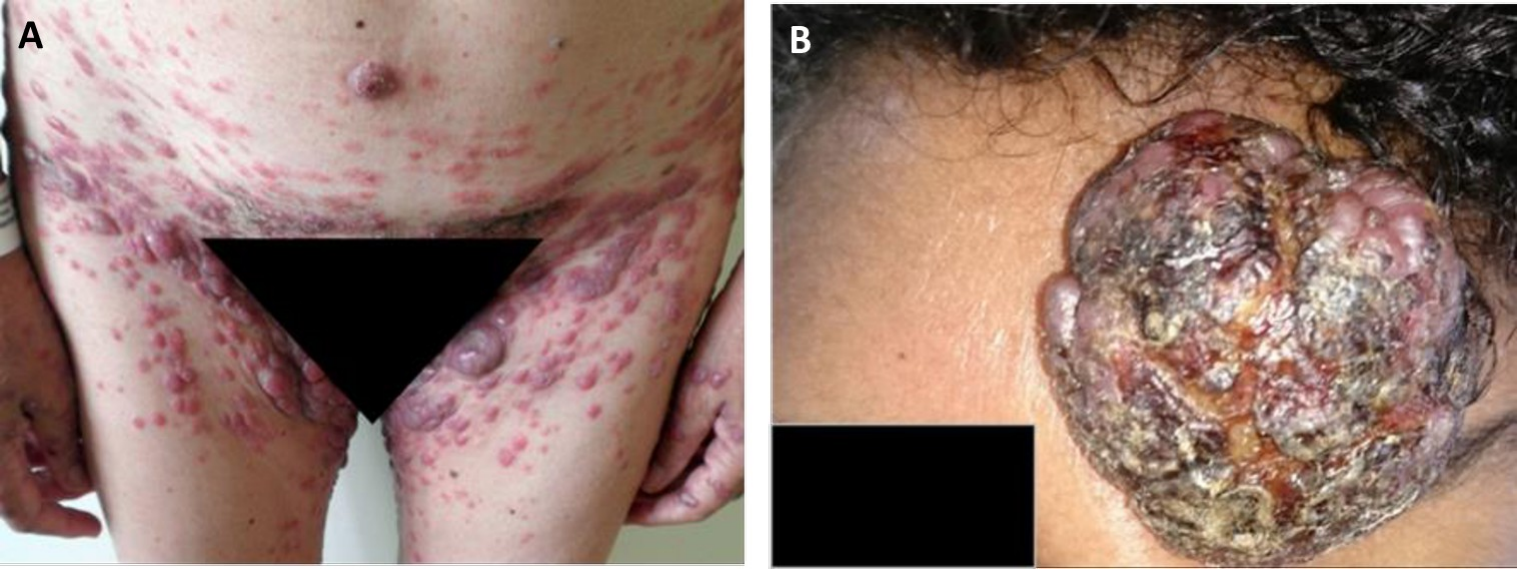
**ATLL/PCT cases: A.** A 57 years-old female with numerous nodules on her body also affecting genitalia, inguinal regions, thighs and hands, with the confluence of some nodules. **B**. A 36 years-old female with a huge tumor on the front.

All the patients were Brazilian and 97% were from Bahia. The median age at diagnosis was 49 years (range 39–63), with a slight female predominance (53.1%). African descendants made up 85.3% and fair-skinned 14.7%. The median time from symptoms onset to diagnosis was 6 months (2–12). Of the 143 cases, 50 (35.0%) were classified as acute, 35 (24.5%) as smoldering, 29 (20.3%) as lymphoma, 23 (16.0%) as chronic, six (4.2%) as PCT/ATL. Sixty-seven percent of the patients had skin lesions at diagnosis. The more frequent skin lesions were plaques (31.1%), followed by multipapular (23,3%), erythroderma (19.0%), nodulotumoral (20.0%) and other lesions (6.6%). PCT lesions were usually identified as multiple nodules or as isolated tumors at the first visit (Fig 1A and 1B) and they tended to grow rapidly.

The distribution of the different clinical manifestations concerning the different clinical disease forms is shown in Table 1. Lymphadenopathy, hepatomegaly, splenomegaly, involvement of more than two organs, and high LDH levels were more frequently observed in the acute, lymphoma, and chronic forms. Bone marrow involvement occurred more frequently in the acute than in the lymphoma type and was absent in the smoldering form and PCT/ATLL. Hypercalcemia was more frequently observed in patients with acute ATLL compared to patients with the lymphoma form. One patient with widespread cutaneous nodules and tumors was included as an acute form only because she had hypercalcemia (11,6 mg/dL). She had no extracutaneous involvement and no lymphocytosis. Three blood smears carried out showed 2% of abnormal cells. This low percentage of abnormal cells was confirmed by flow cytometry (CD4+, CD25+, CD7- with decreased CD3 expression).

**Table 1:** Characteristics of 143 patients with adult T-cell leukemia/lymphoma according to the clinical types.

Characteristics	Total (n = 143)	Smoldering (n = 35)	chronic (n = 23)	PCT (n = 6)	Lymphoma (n = 29)	Acute (n = 50)
	Number of patients (%) or number of patients/number of patients with available data (%)					
Female sex	76 (53.1)	17 (48.6)	13 (56.5)	4 (66.7)	17 (58.6)	25 (50.0)
Age ≥50 years	70 (49.0)	18 (51.4)	11 (47.8)	2 (33.3)	17 (55.2)	22 (44.0)
Lymphadenomegaly	81 (56.6)	0	14 (60.9)	0	29 (100)	38 (76.0)
Hepatomegaly	34 (27.8)	0	3 (13.0)	0	9 (31.0)	22 (44.0)
Splenomegaly	43 (30.0)	0	2 (8.7)	0	11 (37.9)	30 (60.0)
>2 involved organs	54 (37.8)	0	5 (21.7)	0	16 (55.2)	33 (66.0)
Lymphocytosis	72 (50.3)	0	23 (100)	0	0	49 (98.0)
Hypercalcemia	40 (28.0)	0	0	0	8 (27.6)	32 (64.0)
LDH >2 x UNV	46 (32.2)	0	0	0	10 (34.5)	36 (72.0)
Skin involvement	96 (67.1)	35 (100)	17 (73.9)	6 (100)	11 (37.9)	27 (54.0)
BM involvement^1^	38/99 (26.6)	0/18 (0)	5/15 (33.3)	0/6 (0)	6/25 (24.0)	27/35 (77.1)
MF pattern^2^	36/126 (28.6)	24 (68.6)	9 (42.9)	0	0	3 (8.6)
PTCL-U pattern^2^	85/126 (67.5)	10 (28.5)	11 (52.4)	6 (100)	27 (93.1)	31 (88.6)
ALCL pattern^2^	5/126 (3.9%)	1 (2.9%)	1 (4.8%)	0	2 (6.9%)	1 (2.9%)
Small/medium cells^2^	65/126 (52.0)	33 (94.3)	13 (61.9)	2 (33.3)	3 (10.3)	14 (40.0)
Proliferative index <20%^3^	44/118 (37.3)	26 (74.3)	9 (45.0)	0	1 (4.2)	5 (15.1)
HAM/TSP	19 (13.3)	12 (34.3)	3 (13.0)	0	1 (0.5)	3 (6.0)

PCT: Primary cutaneous tumoral; LDH: lactate dehydrogenase; UNV: upper normal value; BM: bone marrow; MF: mycosis fungoides; PTCL-U: Peripheral T-cell lymphoma unspecified; ALCL: Anaplastic large-cell lymphoma; HAM/TSP: HTLV-1 associated myelopathy/ tropical spastic paraparesis; ^1^Data unavailable for 44 patients who did not undergo bone marrow biopsy. ^2^Data is unavailable for 17 patients who did not have a biopsy performed.

Fifteen ATLL patients (10.5%) progressed to acute ATLL (10 chronic, two lymphomas, two PCT, and one smoldering patient). Another eight patients with the smoldering form progressed to other forms of ATLL (seven to chronic and one to PCT) after a median period of 24 months (range 2 to 108 months).

### Anatomopathological features

**Fig 2:**
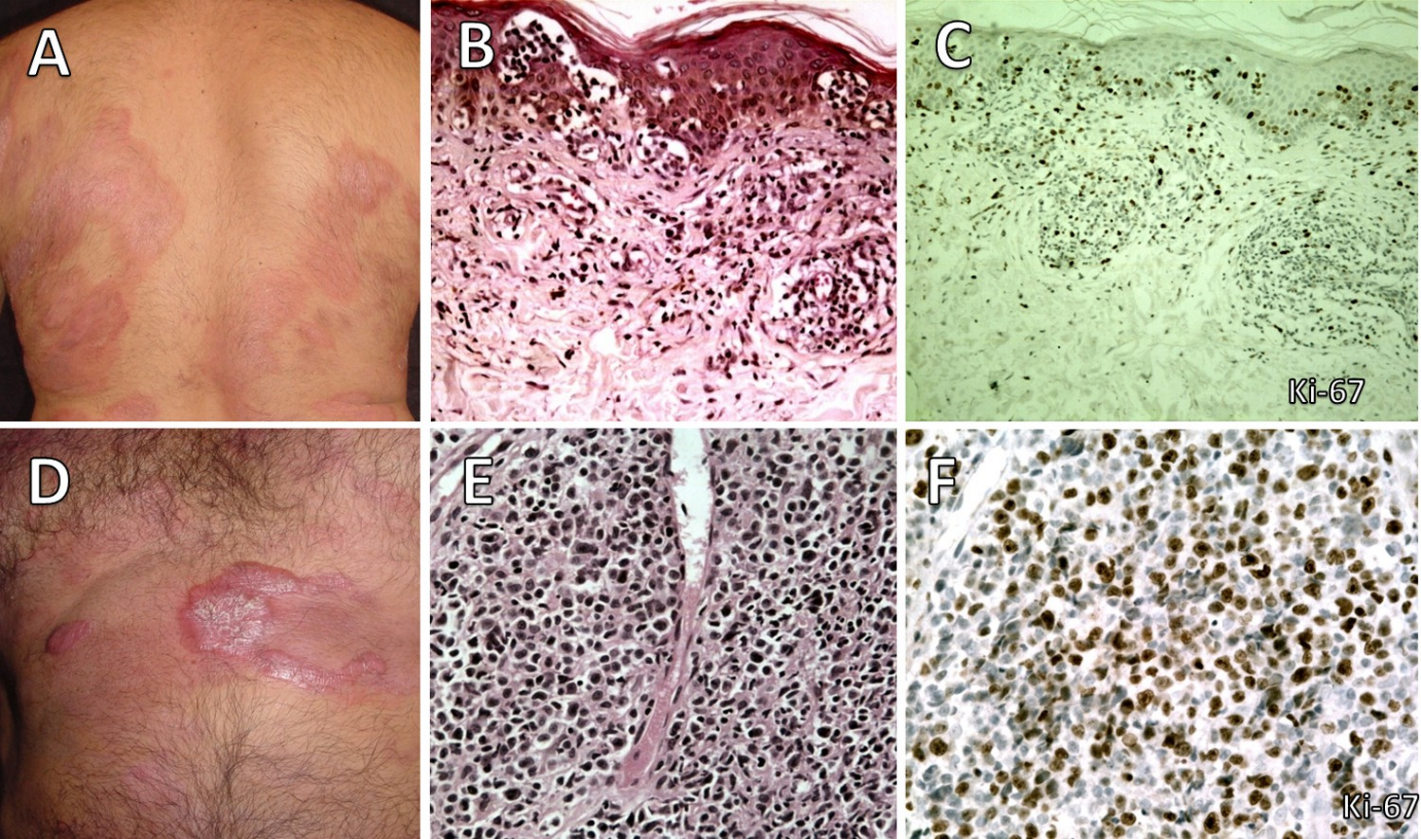
Forty-six years old men with erythematous plaques. **A.** Diagnosis of smoldering adult T-cell leukemia/lymphoma with a microscopical pattern of mycosis fungoides. **B.** Mycosis fungoides pattern with Pautrier’s microabscesses and infiltration of small atypical lymphocytes in the superficial dermis (HE, x160). **C.** Low proliferative index (Ki-67, x160). **D**. Clinical transformation to primary cutaneous tumoral form. **E**. Microscopically, pleomorphic cells infiltrate the dermis and subcutaneous tissue (HE, x400). **F.** High proliferative index of the large cells (Ki-67, x400).

The anatomopathological study of the biopsies revealed that 67.5% of them had the morphology of peripheral T-cell lymphoma with an unspecified pattern (PTCL-U), 28.6% had the mycosis fungoides (MF) pattern, and 3.9% had a morphology of anaplastic large cell lymphoma (ALCL). In the MF pattern, the presence of small/medium cells and a proliferative index ≤ 20% predominated in the smoldering and chronic forms, while in the PTCL-U pattern, the presence of large cells and a proliferative index >20% predominated in the PCT, lymphoma and acute forms. One patient was an example of Hodgkin-like ATLL, but for statistical purposes, she was included in the PTCL-U group. The progression of a case with the smoldering form to the PCT form corresponded histologically to a change from an MF pattern to a PTCL-U pattern (Fig 2). In the MF pattern, the dermal infiltrate consisted of small or medium-sized cells with epidermotropism of varied intensity, linearly infiltrating the basal layer, forming Pautrier’s abscesses (Fig 2B) or configuring a pagetoid distribution with a low proliferative index (Fig 2C). The PCTL-U pattern showed large malignant cells (Fig 2E) and a high proliferative index (Fig 2F). In skin biopsies, large cells infiltrated the dermis and sometimes the subcutaneous tissue.

### Other associated manifestations related to the HTLV-1

Nineteen (13.3%) patients also presented HAM/TSP, whose signs and symptoms began before the onset of ATLL. HAM/TSP was mainly observed in smoldering form (Table 1). Another three patients had infective dermatitis associated with HTLV-1 (IDH). Two of these IDH patients progressed to ATL in adolescence when they were 13 and 15 years old (at the end of the study follow-up, they were 16 and 20 years old). The third IDH patient had the adult form of IDH and developed ATLL 19 years after IDH onset.

### Survival of ATLL patients

**Fig 3:**
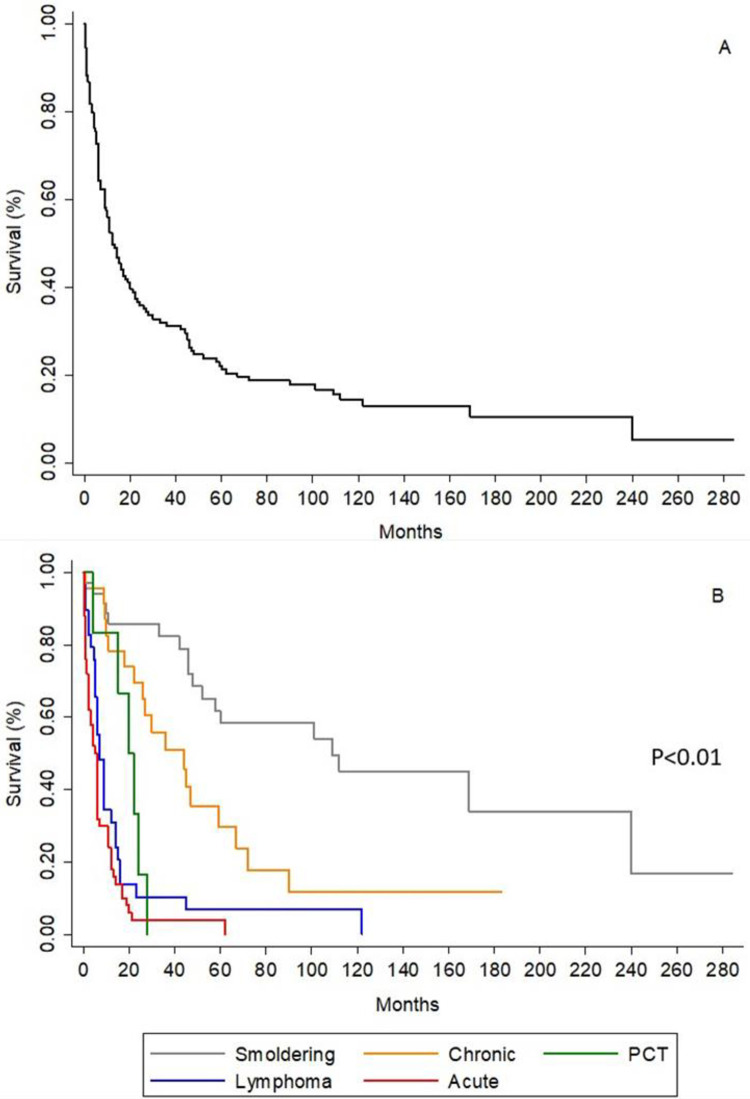
Kaplan-Meier survival estimates. A. Representing the overall survival curve. B. Representing the survival curve for each clinical type.

The overall MST was 12 months (interquartile range 5–45), and the estimated survival rates at two, five, and ten years were 37.1%, 24.5%, and 18.9%, respectively. The MST according to the clinicopathological characteristics is shown in Table 2. Only 24 of the 143 patients (17%) were alive at the end of the study follow-up (17 smoldering, five chronic, one lymphoma, and one acute patient). Of the 18 patients with the smoldering form who died, 13 died from causes unrelated to ATLL, such as infections or other diseases. Kaplan-Meier survival estimates representing the overall survival curve and the survival curve for each clinical type were shown in Fig 3A and 3B, respectively.

**Table 2:** Survival time of 143 patients with adult T-cell leukemia/lymphoma, according to clinicopathological characteristics.

Characteristic	Number of patients	Median [IQR] survival time^1^	Number of P-Y of follow-up	Number of deaths	Mortality rate per 100 P-Y	Hazard ratio (95% CI)	P value
**Sex**							**0.59**
Female	76	15 [5–47]	2,516.3	62	2.5	1	
Male	67	9 [4–58]	2,340.6	57	2.4	0.91 (0.63–1.30)	
**Age group**							**0.28**
<50 years	73	20 [6–48]	2,850.0	62	2.2	1	
≥50 years	70	9 [2–44]	2,006.8	57	2.8	1.22 (0.85–1.75)	
**Clinical types**							**<0.01**
Smoldering	35	109 [46–240]	2825.5	18	0.6	1	
Chronic	23	44 [18–67]	1046.5	18	1.7	2.28 (1.16–4.46)	
PCT	6	20 [15–24]	113	6	5.3	5.10 (1.93–13.43)	
Lymphoma	29	7 [5–14]	470.8	28	5.9	6.89 (3.67–12.96)	
Acute	50	5 [2–11]	401.1	49	12.2	11.23 (6.08–20.76)	
**Histopathology** ^**2**^							**<0.01**
MF	36	39 [14–82]	1869.0	24	1.3	1	
ALCL	5	17 [7–52]	265.0	4	1.5	1.29 (0.45–3.74)	
PTCL	85	10 [4–24]	2483.3	76	3.1	2.10 (1.32–3.34)	
**Proliferation degree** ^**3**^							**<0.01**
≤20%	44	58 [11–169]	2900.0	30	1.0	1	
>20%	74	9 [5–22]	1464.8	66	4.5	2.85 (1.80–4.52)	
**Cell size**							**<0.01**
Small	65	42 [9–109]	3194.0	48	1.5	1	
Large	61	9 [4–14]	1423.3	56	3.9	2.04 (1.37–3.03)	
**Skin involvement**							**0.01**
No	47	7 [5–17]	913.1	42	4.6	1	
Yes	96	19 [5–67]	3943.8	77	2.0	0.61 (0.41–0.89)	
**HAM/TSP**							**0.01**
No	124	11 [5–44]	3786.3	107	2.8	1	
Yes	19	47 [9–]	1070.5	12	1.1	0.49 (0.27–0.89)	
**Lymphadenopathy**							**<0.01**
No	62	48 [12–169]	3689.0	40	1.1	1	
Yes	81	6 [3–15]	1167.8	79	6.8	3.76 (2.49–5.67)	
**Hepatomegaly**							**<0.01**
No	109	17 [6–62]	4343.8	85	2.0	1	
Yes	34	6 [2–14]	513.0	34	6.6	2.23 (1.49–3.34)	
**Splenomegaly**							**<0.01**
No	100	22 [6–90]	4232.6	76	1.8	1	
Yes	43	6 [2–12]	624.3	43	6.9	2.41 (1.64–3.55)	
**Bone marrow involvement** ^**4**^							**<0.01**
No	61	20 [9–58]	2853.8	52	1.8	1	
Yes	38	6 [2–14]	418.8	36	8.6	2.61 (1.67–4.10)	
**Lymphocytosis**							**<0.01**
No	71	23 [7–112]	3421.3	53	1.5	1	
Yes	72	7 [2–22]	1435.6	66	4.6	2.03 (1.40–2.94)	
**Number of involved organs**							**<0.01**
≤2	89	27 [9–109]	4162.3	65	1.6	1	
>2	54	6 [2–12]	694.5	54	7.8	3.05 (2.09–4.45)	
**Hypercalcemia**							**<0.01**
No	103	23 [9–90]	4527.5	80	1.8	1	
Yes	40	5 [2–11]	329.3	39	11.8	3.45 (2.27–5.22)	
**LDH**							**<0.01**
Up to 2x UNV	97	26 [9–90]	4432.3	75	1.7	1	
>2x UNV	46	6 [2–11]	424.6	44	10.4	3.39 (2.26–5.10)	

IQR: Interquartile range; P-Y: person-years; PCT: primary cutaneous tumoral; PTCL-U: peripheral T-cell lymphoma unspecified; MF: mycosis fungoides; ALCL: anaplastic large cell lymphoma; HAM/TSP: HTLV-1 associated with myelopathy/tropical spastic paraparesis; LDH: lactic dehydrogenase; UNV: upper normal value

^1^ In months

^2^ Data unavailable for 17 patients who did not have a biopsy performed

^3^Data unavailable for 8 patients for whom proliferative index evaluation (Ki-67 index) was not performed

^4^ Data unavailable for 44 patients who did not undergo bone marrow biopsy.

### Bivariate and multivariable analysis

Bivariate analyses revealed that the smoldering form had a statistically significantly better prognosis compared to all the other forms (Table 2 and Fig 3B). Cases with lymphadenopathy, bone marrow involvement, hepatomegaly, splenomegaly, involvement of two or more organs, lymphocytosis, high LDH levels, and hypercalcemia had a poor outcome (Table 2).

Regarding the anatomopathological characteristics, the bivariate analyses showed greater survival in patients with MF patterns, small/medium-sized cells, and proliferative index ≤ 20%.

The final multivariable model, which retained only the variables associated with survival at a P<0.05, identified independent factors associated with poor prognosis: the clinical forms the presence of lymphadenopathy, and the presence of skin lesions (Table 3). Interestingly, skin involvement had been associated with a better outcome in the bivariate analysis, before adjusting for clinical form and the presence of lymphadenopathy.

**Table 3:** Clinical characteristics associated with survival time for 143 patients with adult T-cell leukemia/lymphoma in a multivariable analysis.

Characteristic	Number of patients	Median (IQR) survival time^1^	Number of P-Y of follow-up	Number of deaths	Mortality rate per 100 p-y	Crude hazard ratio (95% CI)	Adjusted hazard ratio (95% CI)
**Clinical types**							
Smoldering	35	109 [46–240]	2825.5	18	0.6	1	1
Chronic	23	44 [18–67]	1046.5	18	1.7	2.28 (1.16–4.46)	1.74 (0.80–3.79)
PCT	6	20 [15–24]	113	6	5.3	5.10 (1.93–13.43)	5.83 (2.18–15.55)
Lymphoma	29	7 [5–14]	470.8	28	5.9	6.89 (3.67–12.96)	4.85 (1.95–12.04)
Acute	50	5 [2–11]	401.1	49	12.2	11.23 (6.08–20.76)	9.69 (4.41–21.32)
**Skin involvement**							
No	47	7 [5–17]	913.1	42	4.6	1	1
Yes	96	19 [5–67]	3943.8	77	2.0	0.61 (0.41–0.89)	1.62 (1.04–2.52)
**Lymphadenopathy**							
No	62	48 [12–169]	3689.0	40	1.1	1	1
Yes	81	6 [3–15]	1167.8	79	6.8	3.76 (2.49–5.67)	2.22 (1.22–4.04)

IQR: Interquartile range; P-Y: person-years; PCT: primary cutaneous tumoral

^1^In month.

## Discussion

We evaluated 143 patients with ATLL, classified at diagnosis by Shimoyama’s classification. However, patients classified as smoldering in the original Shimoyama’s classification were subdivided into two groups: patients with the smoldering form and patients with the PCT/ATLL, based on the presence of cutaneous nodules or tumors in the last group. In a smaller cohort of ATLL patients in Bahia, with less follow-up, we had previously observed that PCT patients’ clinical manifestations and survival were quite different from smoldering patients without skin nodules or tumors [7,15]. Here, we also found a large difference in the MST between patients with smoldering ATLL and patients with PCT/ATLL (109 vs. 20 months, respectively). PCT/ATLL corresponds to the cutaneous tumoral ATLL reported by Johno in 1992 [20]. In the last ATLL International Consensus Meeting Report, PCT was named an extranodal primary cutaneous variant of the lymphoma type and was considered an aggressive ATLL type [9].

Our cohort comprised Brazilian patients, 97% of them from the state of Bahia, situated in Northeastern Brazil. About a quarter of the patients had the smoldering form. Some studies on ATLL in Japan and the Americas use the original Shimoyama’s classification, but they included only a few patients with the smoldering form [8,10–12,21,22]. The greater number of smoldering patients in the current study probably occurred because dermatologists were part of our clinical team.

One of our cases was challenging; the patient had widespread cutaneous nodules and tumors, without extracutaneous involvement and lymphocytosis and with only 2.1% of abnormal cells in peripheral blood, and immunophenotyping showed only 2.1% of abnormal cells CD4+. CD25+, CD7- with decreased CD3 expression. It was included in the acute form due to hypercalcemia (11,6 mg/dL), according to the definition of Shimoyama’s classification.

The current observation of a statistically significant difference in the survival of patients with the smoldering and the PCT/ATLL, both by bivariate and multivariable analyses, shows that it is essential to differentiate the smoldering form from PCT/ATLL to determine prognosis and treatment better. It was also observed by multivariable analysis that, acute, lymphoma and PCT/ATLL had a worse prognosis than the smoldering form, regardless of the presence of lymphadenopathy or skin involvement.

The MST of patients with the smoldering form in the present study (109 months) was much longer than that found in a nationwide survey in Japan (55 months) [8]. It was also higher than that found by Takasaki et al [23] in Nagasaki, Japan, who evaluated 90 cases of indolent ATLL (smoldering and chronic) and showed that the MST of smoldering ATLL was only 36 months, even lower than that observed for the chronic form (63 months). Differences in environmental and genetic factors may explain the better outcome for patients with the smoldering form in Brazil. Still, we must consider that, in Japan, patients with PCT, an aggressive form of ATLL, were included in the smoldering type, an indolent form of ATLL [23,24].

Considering the better prognosis of the smoldering form in Brazil, it is important to note that only one (0.7%) of our patients with the smoldering form progressed to the acute form while in Nagasaki, Japan, from 25 smoldering patients, 15 (60%) died of acute ATLL [23]. Besides, in Kyushu, Japan, the risk for smoldering patients progressing to acute ATLL ranged from 20% to 42% [24].

Our study’s overall frequency of deaths was very high, with only 17% of the patients surviving to the date of study censorship, probably due to the predominance of the acute and lymphoma types. The MST of patients with the acute and lymphoma types was shorter than that observed in Japan, possibly due to the more effective chemotherapy and allo-HSCT employed in that country [25]. Among our patients, only one, with acute disease underwent an allo-HSCT.

ATLL was associated with HAM/TSP, a disabling form of myelopathy, in 13.3% of our patients. In Chile, Cabrera et al. [26] also found a high frequency of HAM/TSP among patients with ATLL (27%), predominating among those with the smoldering form. They suggested that genetic and/or environmental factors could explain this high frequency, but they also attributed it to a more accurate neurological evaluation of their ATLL patients. However, in Jamaica, a study found that only 2.4% of patients with ATLL had HAM/TSP [21]. In Japan, this association is also rare [27].

In the current study, three cases of ATLL emerged during IDH; two in the juvenile form of the disease (the patients were 12 and 19 years old) and one in the adult form. The progression of IDH, an infective and recurrent dermatitis, to ATLL has been previously demonstrated [18,28,29].

Our bivariate analysis found that skin involvement was related to a better outcome. However, in the multivariable analysis, the result was the opposite. A better prognosis for patients with cutaneous involvement in the bivariate analysis can be explained by the fact that 36.5% of these patients were of the smoldering type, an indolent form of ATLL with greater survival rates [8]. The adjustment obtained with the multivariable analysis removed the confounding effect between skin lesion and the clinical type. It made it possible to identify that skin involvement was an independent prognostic factor for earlier death in patients with ATLL.

The histopathology of ATLL is non-specific and its histopathological patterns are indistinguishable from those of other peripheral T-cell lymphomas [15]. Without the knowledge that the patient is a carrier of HTLV-1, an examining pathologist will likely diagnose MF, PTCL-U, or ALCL, the most frequent entities that mimic ATLL. By bivariate analyses, patients with the MF pattern had statistically higher survival than those with PTCL-U, a finding not previously observed (Table 2). The bivariate analysis also found a better survival of ATLL with small/medium cells and proliferative index < 20%, an observation previously demonstrated by another study [7].

Based on our findings, we recommend that, at the time of diagnosis, more emphasis should be given to the recognition of the PCT cases considering that this manifestation has a worse prognosis when compared to smoldering ATLL and should be managed differently. Besides, it is crucial not only to recognize but also to report cases of PCT/ATLL to characterize this disease phenotype. Our idea is that PCT/ATLL should be considered another clinical form of ATLL, not a variant of the lymphoma type. Limitations of our study were the relatively small sample size concerning other studies and the impossibility of testing for soluble IL-2R. However, considering that there are still few published cases in Brazil, and this is a prospective single-center study consisting almost exclusively of Afro-Brazilian subjects, we believe that our findings may be helpful for a better understanding and knowledge of ATLL.

## Supporting information

S1 Data Data bank containing all data analysed in this manuscript. (XLS)

S1 Text Data bank dictionary. (DOCX)
