# Supplementary material for: The importance of the clinical classification of adult T-cell leukemia/lymphoma (ATLL) in the prognosis
Source: PLoS Negl Trop Dis. 2022 Oct 19;16(10):e0010807. doi: 10.1371/journal.pntd.0010807 (PMC9581356; doi:10.1371/journal.pntd.0010807)
Supplement: S1 Text — (DOCX) [file pntd.0010807.s002.docx]

DICTIONARY OF THE VARIABLE NAMES OF THE DATA BANK .Paper: “The importance of the clinical classification of adult T-cell leukemia/lymphoma (ATLL) in the prognosis”

| VARIABLE NAME (as displayed in the bank) | DESCRIPTION | LEGENDS |
| --- | --- | --- |
| Clinform | Clinical forms | 1: smoldering  2: chronic  4: primary cutaneous tumoral (PCT)  5: lymphoma  6: acute |
| gender | Gender of participants | 1: male  2: female |
| Age gr | Age groups | 1: < 50 years  2: ≥ 50 years |
| Duration | Duration of disease (time from symptoms onset until diagnosis) | in months |
| HAM/TSP | HTLV-1-associated myelopathy/ tropical spastic paraparesis | 1: without myelopathy  2: with myelopathy |
| lymphadeno | Lymphadenomegaly | 1: without  2: with |
| hepato | Hepatomegaly | 1: without  2: with |
| spleno | Splenomegaly | 1: without  2: with |
| BM | Histopathological exam of bone marrow biopsy | 0: cases without biopsy  1: not involved  2: involved |
| Involv.org | Number of involved organs | 1: Two or less  2: > 2 |
| Lympho | Lymphocytosis (> 4x 10^9^/l) | 1: without  2: with |
| hypercal | hypercalcemia | 1: without  2: with |
| Skin | Skin lesion | 1: without  2: with |
| LDH | LDH values | 0: normal  1: up to 2x the normal value  2: > 2x the normal value |
| Histodiag | Histopathological diagnosis | 0: cases without biopsy  1: Mycosis fungoides  4: Anaplastic large cell lymphoma  5: PTCL |
| cells | Cells size | 0: cases without biopsy  1: small/medium  2: large |
| PI | Proliferative index (Ki-67) | 0: not evaluated  1: ≤ 20%  2: > 20%  3: cases without biopsy |
| Survival | Survival intervals were calculated from the date of diagnosis to the date of death or last follow-up | In months |
| Deaths |  | 0: alive  1: deceased |
